# Acceptance of Self-Sampling Among Long-Term Cervical Screening Non-Attenders with HPV-Positive Results: Promising Opportunity for Specific Cancer Education

**DOI:** 10.1007/s13187-019-01608-0

**Published:** 2019-09-14

**Authors:** Sonia Andersson, Karen Belkić, Miriam Mints, Ellinor Östensson

**Affiliations:** 1 Department of Women’s and Children’s Health, Karolinska University Hospital and Institute, Stockholm, Sweden; 2 Department of Oncology-Pathology, Karolinska Institute, Stockholm, Sweden; 3 Claremont Graduate University, Claremont, CA USA; 4 Keck School of Medicine, University of Southern California, Los Angeles, USA; 5 School of Medical Sciences, Faculty of Medicine and Health, Örebrö University, Örebrö, Sweden

**Keywords:** HPV testing, Cervical cancer screening, Selfcollection, Specific knowledge, Leverage points

## Abstract

This study aims to investigate acceptance of vaginal self-sampling for high-risk human papilloma virus (HPV) among long-term screening non-attenders at increased cervical cancer risk and to identify leverage points to promote screening adherence among these women. Forty-three long-term screening non-attenders performed home vaginal self-sampling for HPV, had positive HPV results, and subsequently attended gynecologic examination. Sixteen (37.2%) had high-grade cervical intraepithelial neoplasia (CIN2 or 3), and two had invasive cervical cancer. Forty-one of these women completed a questionnaire concerning Specific Knowledge about HPV, CIN, and cervical cancer, potential barriers to screening and views about self-sampling. Results were compared with 479 women treated for CIN2+ who attended gynecologic follow-up and also performed self-sampling. Significant multivariate predictors of long-term non-attender status compared with referents were low Specific Knowledge, high confidence in self-sampling, and potential barriers—refraining from activity to attend gynecologic examination, needing another's help to attend, and long travel time. Non-attenders citing fear/refraining from gynecologic examination as why they preferred self-sampling significantly more often had lowest Specific Knowledge compared with other non-attenders. All non-attenders could envision themselves doing self-sampling again while only 74% of referents endorsed this statement (*p* = 0.0003). We conclude that HPV self-sampling is an acceptable option for women at increased cervical cancer risk who have been long-term screening non-attenders. Educational outreach to enhance Specific Knowledge about HPV, CIN and cervical cancer is critical. Those non-attenders who explicitly avoid gynecologic examinations need special attention. Trial Registry: Clinicaltrials.gov NCT02750124

## Introduction

Cervical cancer (CC) continues to be a frequent cause of cancer death worldwide, particularly in less developed regions, both within and outside Europe [1]. Through screening and treatment of cervical dysplasia, CC mortality has been markedly reduced. Wherever CC screening programs are in place, CC is preponderantly found in non-participants in these programs [2]. Sweden has an invitational, population-based CC screening program since 1967. In 2010, the participation rate in this program was 73%, with CC mortality the 9th lowest in the European Union [3]. Notwithstanding these achievements, in Sweden, close to 500 women are diagnosed every year with CC from which about 200 women die annually [4].

Long-term infection with high-risk human papilloma virus (HPV) is the causative agent of the nearly all CC. Compared with cytology, primary screening based upon HPV confers 60–70% greater protection against high-grade cervical intraepithelial neoplasia (CIN2+) and invasive CC [5]. Women with negative cytology who test positive for HPV are at increased risk of developing CIN2+ compared with those with negative HPV and cytology [6]. Thus, HPV primary screening is being introduced in Sweden and elsewhere. Besides HPV testing on samples collected by health professionals, women themselves can collect the samples. Recent data indicate the same high level of accuracy on self-collected samples as on those collected by health professionals [7]. In a large-scale randomized Swedish study, repeated self-sampling of vaginal fluid for HPV testing provided twice higher CIN2+ detection rate compared with Pap smear [8]. HPV testing from self-collected samples is increasingly viewed as a viable screening option [9–11], offering an alternative for screening non-attenders and increasing the CIN2+ detection rate in randomized studies [8, 12]. These issues were investigated in 479 women treated and followed up for CIN2+, for whom more intense follow-up is needed compared with the general population [13, 14]. Surprisingly, their Specific Knowledge about HPV, CIN, and CC risk was quite low [14]. The large majority considered HPV self-collection an acceptable option. The question was raised as to whether the high level of acceptability of self-sampling would hold true for women at increased CC risk who had been non-adherent to gynecologic screening [13]. It was suggested that these women might be willing to do self-sampling given the convenience and minimal expense. Within this framework, it is vital to assess their Specific Knowledge about HPV, CIN, and CC.

Herein, we examine these questions, focusing on long-term non-attenders to the national organized cervical screening in Sweden, who performed self-sampling with a positive result for HPV and thereafter attended recommended gynecologic follow-up. We assess their Specific Knowledge and explore reasons why self-sampling is or is not acceptable for them. Detailed comparisons are performed with the 479 women treated and followed up for CIN2+. We thereby seek to identify potential leverage points to promote adherence to cervical screening guidelines among long-term non-attenders at increased CC risk and to glean insights into how to best meet the needs of this vulnerable group.

## Methods

### Setting and Study Design

The study was carried out within the population-based cervical screening program of Stockholm County. A target group of long-term screening non-attenders is compared with women treated and followed up for CIN2+ (referents). Both groups performed self-sampling for HPV. The target group was selected based on testing positive for HPV and attending gynecologic exam thereafter. Questionnaire data were collected among both groups.

### Target Group: Long-Term Screening Non-Attenders with Positive HPV on Self-Sampling Who Subsequently Attended Gynecologic Exam

Approximately 4% of women who were eligible for cervical screening and had received at least 10 annual invitations were considered long-term cervical screening non-attenders. As part of a health services study (Clinicaltrials.gov NCT02750124), 4000 of these women (24.3%) were randomized to receive an HPV self-sampling kit by mail or were invited to order the kit via an eHealth Web application. Further details on the methodology and results of that study are described in Ref. [15]. Step-by-step instructions were provided for taking and preparing the sample, enclosing the kit in a zip-locked bag placed in a pre-marked envelope. The cobas® PCR Female Swab Sample Packet was used for vaginal self-sampling. The women who were invited to participate were told that all data concerning the self-sample would be handled with full confidentiality and that by submitting a self-sample, they consented to the study. The study was approved by the Stockholm Regional Ethical Review Board 2015/1075-31/1. Altogether, 515 (12.9%) of the invited women performed self-sampling. The results were HPV-positive in 63 (12.2%) women, all of whom were referred for gynecologic examination, but only 43 (68.3%) attended. The gynecologic exam included pelvic examination, cytology, colposcopy, and punch biopsy. A questionnaire given to the 43 women at gynecologic examination was completed by 41 (95.3%).

### Referent Group: Patients Treated and Followed Up for High-Grade CIN and Who Had Not Been Long-Term Non-Attenders

These 479 women had been treated by conization for histologically confirmed CIN2 or CIN3 and were followed up at 6-months at the Karolinska University Hospital (KUH), Stockholm [13, 14]. None had been long-term non-attenders to screening. These patients performed vaginal and urine HPV self-sampling at the first 6-month follow-up gynecologic examination at KUH. They received written description of how to use the self-sampling (Qvintip-Approvix AB Uppsala, Sweden) device for collection of vaginal fluid and urine. Self-sampling was done at the clinic restroom. Subsequent to self-sampling and prior to the gynecological examination, the questionnaire was given. Complete anonymity was assured. The Karolinska Ethics Committee (2006/1273-31, 2014/2034-32) approved the study.

### Questionnaire

The questionnaire was in Swedish. (The English-language translation can be accessed through Ref. [13]). The questionnaire included socio-demographic queries, travel time to and from the examination, and related logistic issues: time taken from work, refraining from some activity, and needing another person's help to attend gynecologic exam. The next part focused on knowledge about HPV, CIN, and CC. These knowledge-related results were analyzed in Ref. [14] for the referents. Thereafter, the participant was queried as to how she views her knowledge about HPV, how she would like to be informed about HPV, CC, and prevention, and how she views her own CC risk without regular gynecologic follow-up (on a scale from 10 (highest) to 1 (lowest)). Finally, queries about HPV self-sampling included whether she could see herself performing self-sampling prior to the next gynecologic visit and whether self-sampling was simple to carry out with sufficient information provided. Insofar as she could see herself performing self-sampling, the reasons why were requested. Ratings were given for HPV tests performed by health professionals, HPV self-sampling and Pap smear, as to whether cervical cell changes would be detected, and she would thereby be protected from developing CC (ratings from 10: highest confidence to 1: lowest).

### Data Analysis

We performed univariate data analysis for the target group: long-term screening non-attenders with positive HPV results. Detailed comparisons were made between the target group and the referents. Analyses were primarily carried out using Yates' chi-squared test (or Fisher's exact test used if any expected cell was < 5). Two-sample “*t*” tests were employed for some normally distributed continuous variables and Mann-Whitney tests of continuous variables that were not normally distributed. In Ref. [14], factor analysis was used to generate a six-item Specific Knowledge Scale for the cohort of patients treated for CIN2+. Herein, we analyzed these knowledge-related queries among the target group. Two independent observers (SA, EÖ) performed content analysis on the open-ended inquiries regarding HPV self-sampling. A third observer (KB) reviewed these for consensus as to which items would be incorporated. Via binomial multiple logistic regression, *β* estimates, standard errors, odds ratios (OR), and 95% confidence intervals (CI) were computed for salient independent variables in adjusted models, with the outcome variable: long-term non-attender to cervical screening with HPV-positive findings from self-collected samples.

## Results

### Clinical Characteristics and Age of the Two Groups

Of the 43 women in the target group, on cone histology fourteen (32.5%) were diagnosed with CIN2 or CIN3, two (4.6%) had invasive cervical cancer, and 27 (62.8%) had normal findings or CIN1. All were age 34 or above (since by definition, non-attenders were women who had not attended cervical screening for at least 10 years) and the majority were over age 40. As described in Ref. [14], among the referents, 37.1% were below age 31. The mean age of the non-attenders was 44.5 ± 8.0 versus 35.0 ± 8.8 for the referents (2-sample “*t*” test, *p* = 0.000).

### Specific Knowledge About HPV, CIN, and Cervical Cancer Among Non-Attenders Versus Referents

**Fig. 1:**
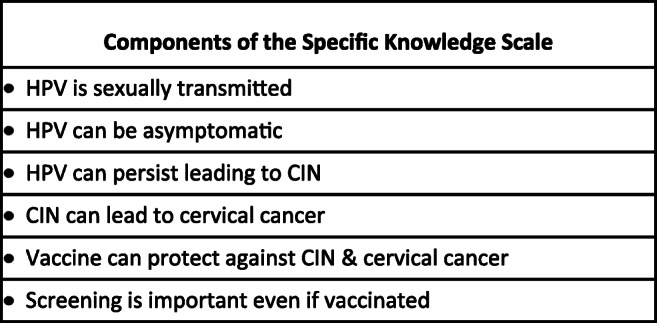
The six components of the Specific Knowledge Scale. High-risk human papilloma virus (HPV), cervical intraepithelial neoplasia (CIN)

Figure 1 displays the six components of the Specific Knowledge Scale. For both the non-attenders and referents, 5 of the 6 components had |factor loadings| > 0.65. The sixth component (screening is important even if vaccinated) showed somewhat lower factor loadings, but was included in the Specific Knowledge Scale because of its clinical/public health importance [14]. The overall percentage of explained variance is somewhat lower in the non-attenders (46.9%) versus 58.3 for the referents. Table 1 indicates that a significantly larger percentage of the non-attenders (41.5%) had the lowest Specific Knowledge scores (0 or 1) compared with the referents (11.9%). Borderline significantly fewer of the women who were non-attenders considered their knowledge about HPV to be good.

**Table 1:**
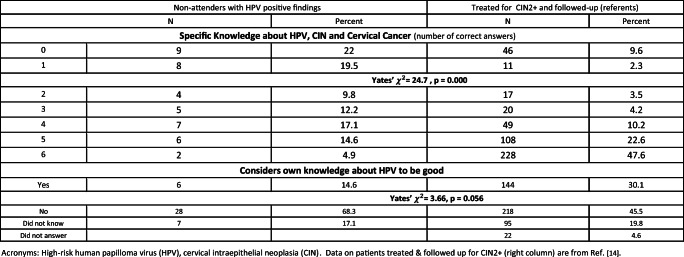
Specific Knowledge about HPV, CIN and cervical cancer, and self-assessed adequacy of that knowledge among long-term screening non-attenders with HPV positive findings compared to referents: women treated and followed-up for high-grade CIN

#### Views on HPV Self-Sampling

The non-attenders unanimously endorsed that HPV self-sampling was easy to perform, sufficient information was provided, and they could envision themselves performing self-sampling prior to the next gynecologic examination. These were all significantly higher endorsements compared with the referents (Table 2). The percentage of highest confidence in HPV self-sampled tests (9 or 10 of 10 maximum) was significantly greater among non-attenders. Confidence ratings for HPV testing from samples collected by health professionals, and Pap smears did not differ significantly between the two groups. Further analysis of those who could envision themselves performing HPV self-sampling prior to the next gynecologic examination includes the 41 non-attenders and 353 referents. Except for time/cost-effectiveness, all the self-generated reasons for readiness to perform self-sampling were percentually more often noted by the non-attenders. Most of these differences were statistically significant.

**Table 2:**
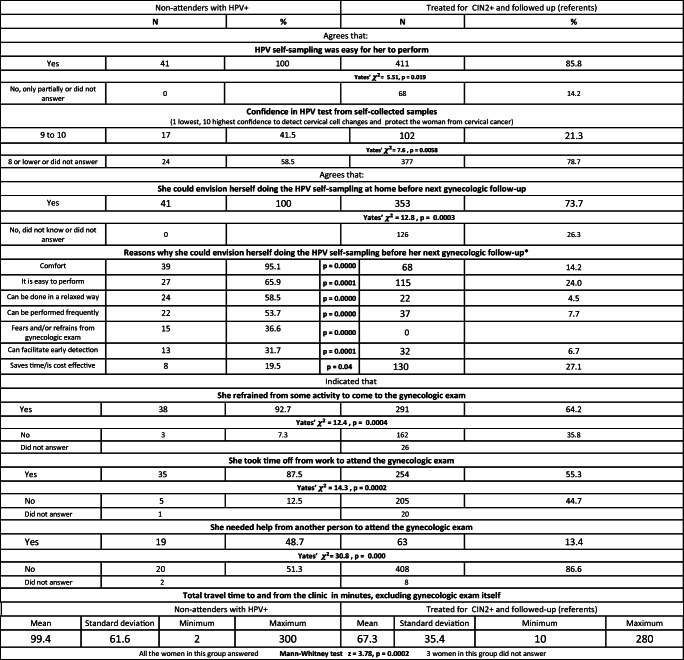
Comparison between long-term screening non-attenders with HPV+ detected from self-sampled specimens versus referents treated for CIN2+ and followed up

*P values given for Yates’ chi-squared or Fisher’s exact test if any expected cell < 5; data on referents from refs. [13, 14]

### Further Univariate Comparisons Between the Non-Attenders Versus Referents

Fewer of the non-attender group had completed university education (39%) compared with referents (58.2%) (Yates' *χ*^2^ = 4.88, *p* = 0.027). The two groups did not differ significantly vis-à-vis annual income, civil status (over half were married or living with their partner in both groups), or employment status (over 70% were employed in both groups). Perceived risk of developing cervical cancer without regular gynecologic follow-up (dichotomized at 7 of 10 maximum) was significantly lower among the non-attenders (Yates' *χ*^2^ = 6.99, *p* = 0.008). Most of the preferred sources of further information about HPV, cervical cancer, and preventive measures were endorsed in similar percentages among the two groups. However, a larger percentage of the non-attenders would like more information via postal mail (Yates' *χ*^2^ = 5.74, *p* = 0.017). Significantly more of the non-attenders stated that they refrained from some activity to attend the gynecologic examination. They had also significantly more often taken time off from work and required another person's help to attend. Total time spent traveling was significantly greater in the non-attenders.

### Multivariate Models Distinguishing Non-Attenders from Women Treated and Followed Up for CIN2+

Table 3 presents the two most complete multiple logistic regression models for distinguishing non-attenders from referents. In model 1, a very low Specific Knowledge score predicted non-attender status. In the second model, high confidence in self-sampling showed significant multivariate association with the non-attender group. In both models, needing another person's help to attend the gynecologic examination and having to cancel/postpone activity showed significant multivariate association with being a non-attender. Having taken 1 h or more for travel also identified non-attenders in both models. This cutpoint was chosen since it was the median time required for both groups combined.

**Table 3:** Multiple logistic regression models identifying non-attenders with HPV+ compared with referents: women treated and followed up for high-grade CIN

	*β* estimate	Standard error	OR	− 95% CI	+ 95% CI	*p*
Model 1 *χ*^2^ = 107, *p* = 0.000						
Specific Knowledge < 2 (of 6 maximum)	1.44	0.47	4.23	1.69	10.6	0.002
Needed another person's help to attend gynecologic exam	1.98	0.46	7.21	2.95	17.6	0.0000
Refrained from activity to attend gynecologic exam	3.27	1.05	26.4	3.30	209	0.002
Total time ≥ 60 min to and from clinic	1.39	0.52	4.03	1.46	11.1	0.007
Age	0.119	0.02	1.13	1.08	1.18	0.000
Education	− 0.183	0.34	0.83	0.43	1.62	NS
Model 2 *χ*^2^ = 102, *p* = 0.000						
Self-sampling confidence rating > 8 (of 10 maximum)	0.93	0.44	2.54	1.07	6.04	0.03
Needed another person's help to attend gynecologic exam	2.08	0.44	8.03	3.37	19.1	0.0000
Refrained from activity to attend gynecologic exam	3.48	1.06	32.6	4.09	259.3	0.001
Total time ≥ 60 min to and from clinic	1.43	0.51	4.17	1.52	11.5	0.006
Age	0.12	0.02	1.12	1.07	1.17	0.0000
Education	− 0.26	0.33	0.77	0.40	1.48	NS

39 non-attenders and 443 women treated and followed up for CIN for whom these data were complete included in the analysis. *NS* statistical non-significance

### Sub-Group Analysis Among the Non-Attenders: Focus on Women Who Explicitly Fear/Refrain from Gynecologic Examination

Fifteen non-attenders explicitly noted fear/refraining from gynecologic examinations as a reason for readiness to do self-sampling. A zero score on Specific Knowledge was more frequent among those who feared/refrained from gynecologic examination (*p* = 0.04, Fisher's exact test) compared with those who did not note this as a reason for self-sampling. Fewer of those who explicitly feared/refrained from gynecologic examination expressed high confidence in Pap smears (*p* = 0.02, Fisher's exact test). Educational level did not significantly distinguish these two sub-groups.

## Discussion

The effectiveness of organized screening programs depends heavily on population coverage; participation is key for preventing cervical cancer. We focus here on a selected group of women at increased cervical cancer risk. Given their long-term non-attendance to the screening program, their increased risk would likely remain undetected had they not performed self-sampling for HPV. Had they not agreed to subsequent gynecologic examination, high-grade CIN would have been missed in a substantial percentage. Of greatest clinical urgency, two cases of invasive cervical cancer would probably not have been found until an even later, more life-threatening stage.

Via the questionnaire, we assess knowledge, attitudes, and needs of these women at increased cervical cancer risk who had declined invitational screening. A very low Specific Knowledge score distinguished them from the referents. This difference remained robust after adjustment for age and education. Notably, over 40% of the target group answered none or only 1 of the 6 specific knowledge questions correctly, compared with fewer than 12% of the referents. In a population-based study from Stockholm [16], poor knowledge about HPV and cervical cancer was associated with low adherence to clinic-based cervical screening. Minimal knowledge about HPV, its relation to cervical cancer, and the importance for screening has also been found among under-screened groups in other countries [10]. Effective strategies, especially via the media, for improving specific knowledge have been reported [17]. Our multivariate model 1 suggests that efforts to improve specific knowledge among long-term non-attenders could be a key leverage point for promoting their participation in screening.

In our previous analyses of the referents, Specific Knowledge scores showed a significant multivariate association with realistic assessment of cervical cancer risk without regular gynecologic follow-up (score ≥ 7) [14]. However, for the target group in this study, Specific Knowledge and self-perceived risk were unrelated (Pearson *r* = 0.08, *p* = 0.63), likely due, in part, to their very low Specific Knowledge. Overall risk among the target group as a whole is lower, since over 60% had normal findings or CIN1, whereas the control cohort all had CIN2+. This difference is a limitation of the present study for comparing the two groups.

Another difference between the two groups is age-related. Since invitational screening in Sweden begins at age 23, the youngest women in the target group were 34 years old. Among the control cohort, this age constraint was not in operation and nearly 40% were 30 or younger. These age differences were fully taken into account in the multivariate analysis, as were differences in educational level.

We anticipated low educational levels among women who explicitly cited fear or other reasons for refraining from gynecologic examination. However, this was not the case. In fact, six of these fifteen women had completed university education. Strikingly, 40% of these women had zero Specific Knowledge scores, suggesting that they actively avoided information about HPV, CIN, and cervical cancer.

The unanimous acceptance of self-sampling and significantly greater percentage of highest confidence in self-sampling compared with referents are also striking findings. These findings, including those of multivariate model 2, suggest that self-sampling could be another leverage point for promoting participation in screening among long-term non-attenders. It should, however, be noted that self-sampling was performed in different settings: at home for the target group versus at the clinic for the referents. Moreover, the referents collected vaginal and urine samples, whereas the target group collected only vaginal samples.

Swedish data show that vaginal self-sampling for HPV is an effective strategy for increasing population coverage [12]. The earlier-cited randomized investigation [8], including all 36,390 women between age 30–49 years within the Uppsala county screening program showed a higher participation rate for self-sampling versus clinic-based cytology (47% versus 39%), with improved pre-cancerous lesion detection. Internationally, as noted, HPV self-sampling is widely accepted, including among screening non-attenders [9–11]. However, to the best of our knowledge, this is the first study to examine self-sampling among very long-term screening non-attenders (> 10 years) with HPV-positive findings, i.e., non-attenders at particularly high risk and who, therefore, would benefit the most from this option.

Practical issues associated with attending gynecologic examination were identified as significant in both multivariate models. Namely, the target group was distinguished from referents by significantly more often requiring another person's help and refraining from activity to attend gynecologic examination, plus more often having traveled over 1 h to and from clinic. These were likely barriers to attending clinic-based screening. Concordantly, among a population-based cohort in Stockholm, a significant association was reported between taking time from work and non-adherence to clinic-based cervical screening [15]. Time constraints, transportation, and taking time from work were identified as barriers to clinic-based screening in other settings [18].

Several women who indicated that they feared/refrained from gynecologic examinations explicitly stated that the reason was having suffered sexual abuse. In a study from southeastern Sweden [19], among 133 women who had been cervical screening non-attenders for 5 years, 22 (16.5%) reported a history of sexual abuse. Among women age 25 or younger, unwanted sexual experiences with genital contact were found to be a risk factor for invasive cervical cancer [20]. Victims of sexual abuse are reportedly at risk for being under-screened or never screened for cervical cancer and other cancers [21–23]. Concordant with our findings, among under- and never-screened populations, an easier test that could be done at home was identified as a key facilitator [23]. Providing accurate information about cervical screening is vital for women who had suffered sexual abuse [21].

The present study yields insight into a unique group of cervical screening non-attenders. Their increased cervical cancer risk associated with HPV-positive findings was identified via self-sampled specimens. Subsequently, these women agreed to attend gynecologic examination and 95% completed the questionnaire. The fact that all but two of these 43 women completed the questionnaire suggests that once these women are under care, they may become more proactive. They could potentially serve as a social norm to encourage their family, friends, and wider network to attend screening, or at the very least, to perform self-sampling. Still, this target group is highly selected and represents a very small percentage of long-term non-attenders. Efforts are needed among the larger remainder of non-attenders. In particular, women of ethnic minority background, especially those not fluent in the host country language, are often non-attenders [24]. A reason why the invitation to self-sampling was not accepted may have been related to language barriers among minority women not fluent in Swedish. Our findings thus cannot be directly generalized to long-term non-attenders, who decline participation in the self-sampling due to language barriers. Recent data suggest that when instructions are presented in the woman's preferred language, in a culturally sensitive manner, self-sampling is well accepted [25]. Such linguistically appropriate, culturally sensitive outreach could be a critical next step for increasing acceptance of self-sampling among long-term non-attenders here in Sweden and elsewhere.

Further studies are needed, focusing on these hardest-to-reach long-term non-attenders. A promising intervention would be to systematically promote self-sampling for HPV within community health programs, concomitant with multi-cultural and multi-linguistic educational efforts through various media. These efforts would likely be most effective if closely coordinated with population-based cervical screening. Therein, knowledge is critical for decision-making regarding screening and for choosing the screening modality that best suits the individual.
